# Effectiveness and safety of auricular acupuncture for psoriasis: A protocol for systematic review and meta-analysis

**DOI:** 10.1097/MD.0000000000032020

**Published:** 2022-11-18

**Authors:** Kaiyuan Xue, Haiyuan Wu, Lidan Jia, Suqing Yang

**Affiliations:** a School of Graduate, Heilongjiang University of Traditional Chinese Medicine, Harbin, Heilongjiang Province, China; b First Affiliated Hospital, Heilongjiang University of Chinese Medicine, Harbin, Heilongjiang Province, China.

**Keywords:** auricular acupuncture, meta-analysis, psoriasis, systematic review

## Abstract

Psoriasis is a chronic, immune-mediated, polygenic skin disease that is common in clinical practice and often develops on the extremities, back, and scalp of patients. In that psoriasis lesions are stubborn and prone to recurrence, it has a serious impact on patients’ quality of life and is detrimental to their physical and psychological health. Auricular acupuncture is one of the traditional Chinese medical treatments, which has the advantages of low adverse effects and simple operation and has been widely used in clinical practice with good efficacy. However, there has been no systematic evaluation of auricular acupuncture in the treatment of psoriasis. This protocol aims to evaluate the effectiveness and safety of auricular acupuncture in patients with psoriasis.

**Methods:**

We will search the following 8 databases including PubMed, MEDLINE, EMBASE, Cochrane Library, CNKI, Wan Fang, VIP, and CBM databases for randomized controlled trials of auricular acupuncture treated psoriasis from their inception to 10 October 2022. We will analyze the data meeting the inclusion criteria with the RevMan V.5.4 software. Two authors will assess the quality of the study with the Cochrane systematic evaluation tool. Treatment effectiveness and the psoriasis area and severity index are defined as the main outcomes, and the additional outcomes include itchy, dermatology life quality index, relapse rate, and adverse events.

**Results:**

This study will review and evaluate the available evidence on the effectiveness and safety of auricular acupuncture for psoriasis.

**Conclusions:**

The results of this study will provide evidence for the effectiveness and safety of treating psoriasis, providing clinicians and patients with appropriate treatment options for this disease.

## 1. Introduction

Psoriasis is a inflammatory disease, often prone to relapse and involves multiple systems, and its pathogenesis is very complex. It is currently believed that its pathogenesis is an immune skin disease mediated by abnormal cellular immunity under the action of multiple factors such as genetics and environment, and is regarded as one of the difficult diseases in dermatology.^[1,2]^ The worldwide prevalence of psoriasis is 1% to 3%, with approximately 100 million people suffering from the disease.^[3]^ The incidence is increasing year by year due to the influence of environmental factors and the increased stress of school and work.^[4,5]^ The typical manifestation of this disease is limited or generalized scaly erythema or plaques, mostly on the head, back, and extremities, or in severe cases all over the body. Pruritus is one of the most common manifestations, and repeated scratching causes the release of more inflammatory mediators, resulting in more intense pruritus, with itchy lesions and scratching in a vicious cycle. Although it does not pose a threat to the safety of patients, it can seriously affect their mental state and quality of life.^[6]^ There is no good treatment that can completely cure psoriasis or control its recurrence.^[7]^ Long-term treatment puts patients under a huge mental and economic burden.^[8]^ The drugs commonly used in clinical practice include retinoic acid, immunosuppressants, and glucocorticoids, but many side effects can occur with the long-term application.^[9–11]^ Auricular acupuncture is a kind of microneedle therapy that stimulates auricular points by acupuncture, moxibustion, or auricular pressure on the auricular distribution. Due to the advantages of long-lasting efficacy and simple operation, auricular acupuncture has become one of the main methods for the treatment of psoriasis. However, there is no systematic evaluation report on the efficacy and safety of auricular acupuncture on psoriasis. Therefore, this study will collect data from randomized controlled trials (RCTs) on auricular acupuncture for psoriasis, objectively evaluate the efficacy and safety of auricular acupuncture for psoriasis, and provide an evidence-based basis for clinical decisions on auricular acupuncture.

## 2. Methods and analysis

### 2.1. Study registration

This protocol has been registered on PROSPERO (registration number: CRD42022369809). We will report it according to the preferred reporting items for systematic reviews and meta-analysis protocols guidelines and meta-analysis protocols.^[12]^

### 2.2. Eligibility criteria

#### 2.2.1. Types of studies.

All RCTs that evaluated auricular acupuncture for psoriasis will be included in this study.

#### 2.2.2. Types of participants.

Patients must meet the following requirements:

1. All patients included in this study must meet the diagnostic criteria for psoriasis;
2. Patients with an age ≥18 years;
3. There will be no restrictions on gender, race, nationality, the severity of disease, or the duration of treatment of the participants.

#### 2.2.3. Type of interventions.

The main intervention in the experimental group will be auricular acupuncture, which also includes trials in combination with medication or other combination therapies. Patients in the control group will be treated with conventional or no treatment.

#### 2.2.4. Types of outcome measures.

##### 2.2.4.1. Primary outcomes.

Treatment effectiveness and the psoriasis area and severity index are defined as the primary outcomes.

##### 2.2.4.2. Secondary outcomes.

Secondary outcomes include the following aspects:

1. Itchy (VAS).
2. Dermatology life quality index.
3. Relapse rate.
4. Adverse events.

### 2.3. Search strategy

We will search national and international articles on auricular acupuncture for psoriasis from PubMed, MEDLINE, EMBASE, Cochrane Library, CNKI, Wan Fang, VIP, and CBM databases. All RCTs in English and Chinese will be included from the establishment of the database to October 10, 2022. In addition, we will also manually retrieve other documents that meet the requirements, such as references, and conference papers. The search strategy for PubMed is shown in Table 1.

**Table 1:** Search strategy for PubMed.

Number	Search terms
#1	Psoriasis [Mesh]
#2	Psoriasis [Title/Abstract]
#3	Psoriases [Title/Abstract]
#4	Psoriatic [Title/Abstract]
#5	Psoria [Title/Abstract]
#6	#1 OR #2 OR #3 OR #4 OR #5
#7	Auricular acupuncture [Mesh]
#8	Auricular acupuncture [Title/Abstract]
#9	Auricular needle [Title/Abstract]
#10	Ear acupuncture [Title/Abstract]
#11	Auricular point sticking [Title/Abstract]
#12	Auricular point [Title/Abstract]
#13	#7 OR #8 OR #9 OR #10 OR #11 OR #12
#14	Randomized controlled trial [Title/Abstract]
#15	Clinical trial randomized [Title/Abstract]
#16	Controlled clinical trial [Title/Abstract]
#17	Clinical trial [Title/Abstract]
#18	Randomized [Title/Abstract]
#19	Randomly [Title/Abstract]
#20	Trial [Title/Abstract]
#21	#14 OR #15 OR #16 OR #17 OR #18 OR #19 OR #20
#22	#6 AND #13 AND #21

### 2.4. Data selection and extraction

#### 2.4.1. Study selection.

**Figure 1.**
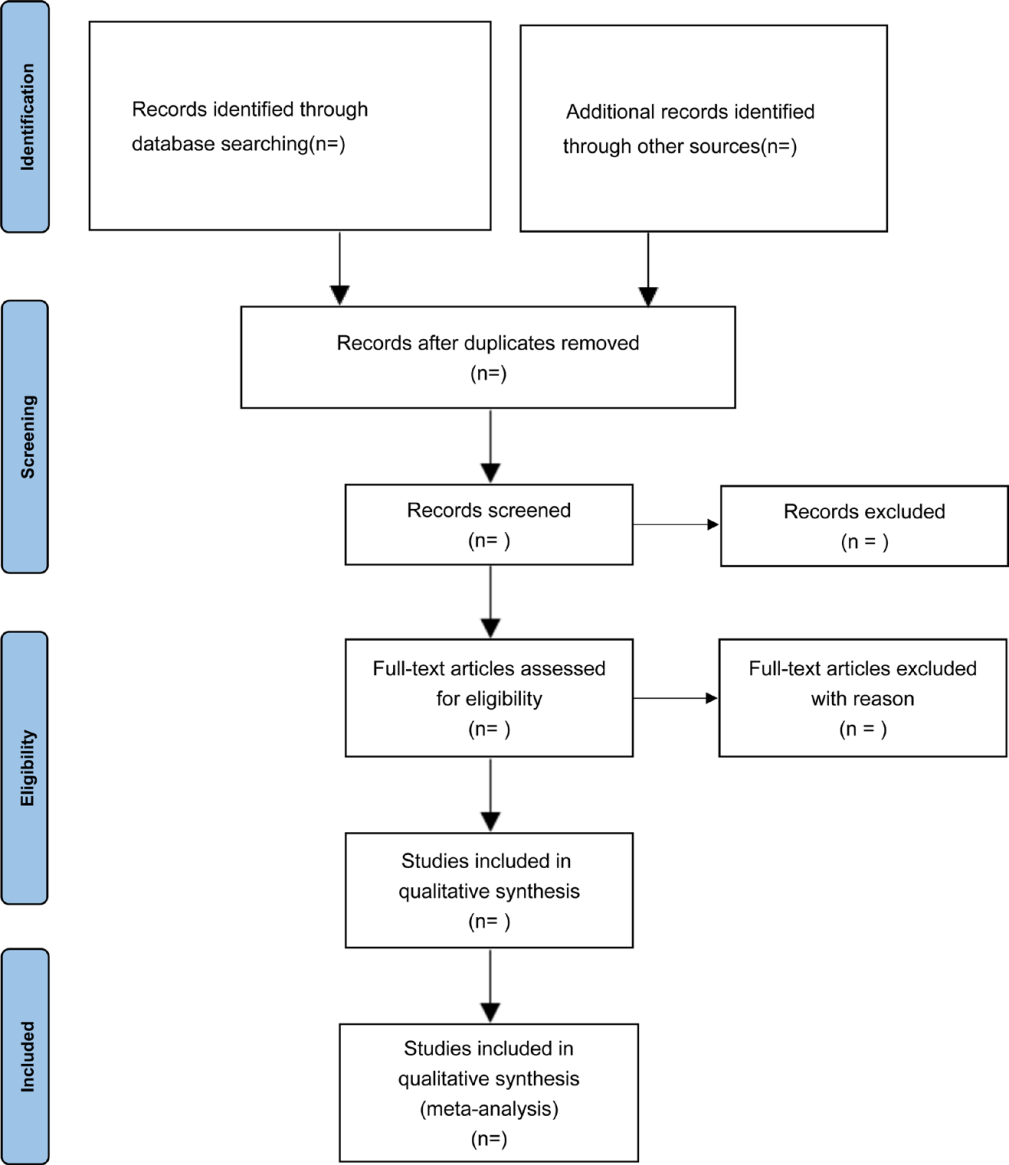
Flow chart of study selection.

Two investigators will independently screen the obtained articles based on the inclusion and exclusion criteria of the review. If the inclusion criteria are met, the titles and abstracts in the literature will be further reviewed and screened. The 2 researchers will then independently read carefully to screen eligible articles. During this process, disagreements between the 2 researchers will be resolved through discussions with a third researcher. The specific screening process is shown in Figure 1.

#### 2.4.2. Data extraction and management.

Two investigators will independently extract data from the selected articles. The extracted data will mainly include the first author, title, country, region, year of publication, sample size, study methods, interventions, patient characteristics, outcomes, and adverse events. Two investigators will enter the extracted data into Microsoft Excel and cross-check them. If there is any disagreement between the 2 reviewers during the screening process, it will be resolved by referring to the original article and discussing it with the third investigator.

#### 2.4.3. Assessment of the risk of bias.

Two researchers will use the Cochrane systematic evaluation tool to assess the risk of bias in included trials independently.^[13]^ The bias tool embodies 7 components: random sequence generation, allocation concealment, blindness of participants and caregivers, blindness of outcome evaluators, incomplete outcome data, selective outcome reports, and other biases. Each item will be assessed on 3 levels: high risk, low risk, or unclear risk. All disagreements will be resolved by discussion with a third investigator to reach a consensus.

#### 2.4.4. Measures of treatment effects.

Assuming that the data are continuous, we will use the mean difference with 95% confidence intervals to represent it. Dichotomous data will be calculated using the dominance ratio of 95% confidence intervals.

#### 2.4.5. Dealing with missing data.

If there are missing data in the article, we will contact the corresponding author via email to obtain the relevant information. If the corresponding author can not be contacted, we will analyze the available data and discuss the potential impact of the missing data on the results of this study.

#### 2.4.6. Data synthesis.

The extracted data will be synthesized and subjected to a meta-analysis in RevMan V.5.4. We will use a fixed-effects or random-effects model based on the results of the heterogeneity test. If the data are not suitable for quantitative analysis, we will provide a descriptive analysis to address this issue.

#### 2.4.7. Subgroup analysis.

If there is significant heterogeneity among all study outcomes, we will perform subgroup analyses and explore the sources of heterogeneity according to study types, intervention methods, treatment frequency, and types of psoriasis.

#### 2.4.8. Sensitivity analysis.

We will perform sensitivity analyses based on sample size, methodological quality, and missing data, and will eliminate low sample tests, low quality tests, or tests with high heterogeneity to improve the accuracy and confidence of the results.

#### 2.4.9. Quality of evidence.

Two investigators will individually apply the Grading of Recommendations Assessment, Development, and Evaluation method to assess the quality of evidence for each outcome. The quality of evidence will be classified as high, medium, low, or very low.^[14]^

## 3. Discussion

Pruritus is an unpleasant sensation with a desire to scratch and is one of the common symptoms of many primary and systemic skin diseases, with an incidence of 62% to 97% in patients with psoriasis.^[15,16]^ Patients with psoriasis have a high frequency and pronounced rash with a long duration, which is not life-threatening but affects aesthetics and skin function and can even lead to anxiety and depression.^[17]^ The clinical treatment of psoriasis is aimed at controlling and stabilizing the disease, reducing adverse effects and improving quality of life. Auricular acupuncture is a long-established traditional Chinese medical treatment method, which is easy to operate, inexpensive and easily accepted by patients. According to Traditional Chinese Medicine theory, auricular acupuncture has the function of unblocking the meridians, adjusting the function of 5 viscera and 6 bowels and regulating the balance of yin and yang. Relevant studies have shown that auricular acupuncture can promote qi and blood circulation to confirm this view.^[18]^ Western medicine believes that auricular acupuncture can prevent and treat diseases by stimulating the nerves on the auricle, which in turn stimulates the cerebral cortex and internal organs and has a bi-directional regulatory effect.^[19]^ Nevertheless, there has been no meta-analysis on the efficacy and safety of auricular acupuncture in the treatment of psoriasis. The results of this study will objectively and comprehensively evaluate the efficacy and safety of auricular acupuncture in the treatment of psoriasis and provide a scientific basis for its clinical application. Meanwhile, this study will also promote the development of auricular acupuncture and the treatment of psoriasis.

## Author contributions

**Conceptualization:** Kaiyuan Xue, Haiyuan Wu, Suqing Yang.

**Data curation:** Kaiyuan Xue, Haiyuan Wu, Lidan Jia.

**Formal analysis:** Kaiyuan Xue, Haiyuan Wu, Lidan Jia.

**Methodology:** Suqing Yang.

**Project administration:** Suqing Yang.

**Writing – original draft:** Kaiyuan Xue.

**Writing – review & editing:** Haiyuan Wu.
